# Experiences of Primary Care Nurse Case Managers in Palliative Care Needs Identification and Complex Chronic Patients’ Referral to Advanced Palliative Care Resources

**DOI:** 10.3390/healthcare14010085

**Published:** 2025-12-30

**Authors:** María Inmaculada Herrera-Gómez, Luz María Iribarne-Durán, María Paz García-Caro, Manuel López-Morales, Ana Alejandra Esteban-Burgos, Rafael Montoya-Juárez

**Affiliations:** 1 Instituto de Investigación Biosanitaria de Granada (ibs. GRANADA), 18012 Granada, Spainmpazgc@ugr.es (M.P.G.-C.); malomorales@ugr.es (M.L.-M.); aesteban@ujaen.es (A.A.E.-B.); rmontoya@ugr.es (R.M.-J.); 2 Centro Nacional de Sanidad Ambiental, Instituto de Salud Carlos III (CNSA-ISCIII), 28222 Granada, Spain; 3 Departamento de Enfermería, Universidad de Granada, 18016 Granada, Spain; 4 Centro de Investigación Mente, Cerebro y Comportamiento, Universidad de Granada, 18071 Granada, Spain; 5 Servicio de Urgencias de Atención Primaria, Sistema Andaluz de Salud, 18013 Granada, Spain; 6 Departamento de Enfermería, Universidad de Jaén, 23071 Jaén, Spain

**Keywords:** palliative care, palliative medicine, terminal care, primary care, prognosis, nursing

## Abstract

**Introduction:** Palliative needs assessment and referral to advanced palliative care resources are fundamental aspects of complex chronic patients’ care. Primary care Nurse Case Managers play a key role in the care of these patients. **Objective:** We aimed to describe the experiences of primary care Nurse Case Managers in palliative care needs identification and complex chronic patients’ referral to advanced palliative care resources. **Method:** This is a qualitative descriptive study with a phenomenological approach. Semi-structured online interviews were conducted with primary care Nurse Case Managers. A thematic analysis was performed using ATLAS.ti software. **Results:** 20 nurses participated, 16 of whom were women, with a mean age of 52.3 years and an average of 15.9 years of experience in primary care. Regarding “Palliative care Needs Assessment”, four sub-themes have been identified: “What do you understand?”, “How do you assess?”, “Difficulties” and “Alternatives” to current palliative care needs assessment. For the “Palliative Care Referral” theme four sub-themes have been identified: “Criteria”, “Tools”, “Difficulties” and “Alternatives” for referral. Discussion: Palliative needs are identified in patients with incurable diseases when there are no curative treatment options and when quality of life must be prioritized. Symptoms, general condition, progression, and comorbidity are assessed. Open interviews and home visits are essential for assessing the social and family context and the home resources available. Barriers identified include the conspiracy of silence, limited training in non-oncological palliative care, and a lack of staff and caregiver’s understanding of illness situation. The presence of difficult symptoms and a limited life expectancy were identified as key criteria for referral to palliative care. The physician’s assessment, the family’s request, and consultation with specialized teams play a key role in prognosis. Barriers include late referrals, lack of a palliative background, inequity in access to resources, and low visibility of the palliative care needs of non-cancer patients. **Conclusions:** Significant challenges remain in identifying palliative needs and referral to specialized resources, highlighting the need to optimize resources, strengthen professional training, and improve coordination between levels of care to ensure quality palliative care.

## 1. Introduction

Palliative care (PC) focuses on improving the quality of life of patients and families facing problems associated with life-threatening illnesses through the prevention and relief of suffering [1]. Although initially focused exclusively on the care of cancer patients, its current scope includes any chronic disease in advanced and terminal stages. According to the World Health Organization (WHO), palliative care should begin at the onset of any life-threatening illness and become more relevant as the patient progresses toward death [1].

The model of Care for People with Complex Needs in Catalonia [2] defines three situations related to the progression of chronic disease: the complex chronic patient (CCP), the advanced chronic patient, and the terminally ill patient. CCPs are chronic patients who have complex health needs arising from the coexistence of chronic diseases, organ or system dysfunction, and factors involving healthcare or social complexity [3]. In the case of advanced chronic patients, palliative care needs (PCN) arise, such as limited life expectancy and increased demands of symptom control, management of therapeutic futility, identification of patient values and preferences, attention to spirituality, and addressing the psychosocial needs of the patient and family [4,5,6].

Healthcare professionals, especially in primary care (PC), must be trained to identify CCPs with PCN. To improve the identification of PCN, it is necessary to have instruments that allow standardization of data collection and facilitate comparison over time, such as the NECPAL CCOMS-ICO [5]. The NECPAL CCOMS-ICO tool is a validated screening instrument designed to support the early identification of individuals with PCNs through a multidimensional, prognostic, and needs-based approach, developed by the Catalan Institute of Oncology. This tool integrates the Surprise Question (“*Would you be surprised if this patient died within the next 12 months?*”) as a screening tool to assess 14 indicators of PCN, including functional, cognitive, and nutritional decline, the presence of dependence, multimorbidity, healthcare resources, and specific indicators of advanced chronic diseases.

On the other hand, another fundamental characteristic of the integrated PC model proposed by both the WHO and the European Association for Palliative Care (EAPC) is the provision of care at two levels: basic and advanced PC [7,8]. Basic PC should be provided by all professionals, in any context (primary care, hospitals, nursing homes) to patients with non-complex PCN. In contrast, specialized PC is provided by specialized resources and services whose main activity is the provision of PC, aimed at patients with high palliative complexity. Some patients will only require basic PC, but others will need to be referred to specialized PC, in complex situations or on a continuous basis until their death. Referral to PC means that specialized PC team professionals must act in coordination with teams providing basic care, especially those in primary care, to provide continuity of care throughout the course of the disease [7]. In the context of the Andalusian Public Health System, the Palliative Care Complexity Diagnostic Tool (IDC-Pal) [9,10] allows the identification of patients who require referral to specialized PC, establishing the level of palliative complexity based on the clinical, psychological, and social factors of the patients.

The role of primary care healthcare professionals is fundamental in identifying palliative needs and referring patients with complex conditions to specialized PC resources. In this regard, primary care nurse case managers (NCM) in Andalusia have the necessary skills to participate in both functions [11]. Although NCMs do not have the competences to refer patients to specialized palliative care resources themselves, they can participate in the discussion about which patients may need these resources and should help gather all the necessary information for this purpose. Nevertheless, the experience of these professionals regarding the identification of palliative care needs and the referral to specialized PC resources is crucial and has not been described in previous research.

This study is part of the INCOPAL project (AP-0209-2019), which aimed to determine the effectiveness of an intervention based on the standardized assessment of the PCN and complexity of CCPs, using the NECPAL CCOMS-ICO and IDC-Pal tools. At the end of the project, it was decided to conduct a qualitative study to describe the experiences of the NCM, both participating and non-participating in the INCOPAL project, regarding the identification of PCN and referral of CCP to specific PC resources.

## 2. Methods

### 2.1. Design

Descriptive qualitative study with a phenomenological approach. This methodological perspective allows for an in-depth exploration and comprehensive understanding of the participants’ experiences in their professional practice, considering their accounts as a primary source of knowledge [12]. Qualitative descriptive studies produced findings closer to the data as given, (or data-near), and are linked to thematic analysis [13].

### 2.2. Selection of Participants

Primary care NCMs with more than three years of experience were selected from centres in all provinces of Andalusia. NCMs who had been part of the INCOPAL project participated were asked to recommend at least one other NCM who had not participated in the project and was not familiar with its objectives or results, using snowball sampling.

### 2.3. Data Collection Instruments and Procedure

A semi-structured interview script (Table 1) was used, developed ad hoc and organized into three blocks: PCN, Referral to PC, and evaluation of the INCOPAL project (this was only answered by project participants). The professionals were contacted by phone or email, agreeing with each one on a date for the interview. All interviews were conducted via Google Meet and recorded on video. The researchers who conducted the interviews had no professional relationship with the interviewees. Authorization for recording and subsequent transcription was obtained at the beginning of each interview. Participants were guaranteed the right to withdraw from the study at any time, without having to justify their decision or suffer any prejudice as a result. The recordings were accessible only to the research team and for the sole purpose of transcribing the results.

### 2.4. Data Analysis

The interviews were transcribed verbatim. A thematic analysis was conducted, following a structured sequence. Prior the analysis, the researchers reflected of their positions and experiences on palliative care needs identification and complex chronic patients’ referral to advanced palliative care resources. First, each transcript was read thoroughly to familiarize with the content. Subsequently, a second, more detailed reading was conducted, assigning codes to the most relevant pieces of information. The themes and sub-themes were determined by the interview questions, while the codes were identified inductively. Two researchers discussed and agreed on the codes. A third researcher participated in the discussion in case agreement could not be reached on the inclusion of a code. The researchers considered that theoretical saturation had been reached, given that no new codes appeared in the last interviews conducted. The entire analysis was performed using ATLAS.ti 9.1.3 software (Berlin, Germany). This study has been approved by the Research Ethics Committee of the Andalusian Public Health System.

## 3. Results

As shown in Table 2, 20 NCMs from Andalusia participated in the study. 9 NCMs (45%) participated in the INCOPAL project, while 11 (55%) did not. Of the total number of participants, 4 were men (20%) and 16 were women (80%). The mean age was 52.3 years (SD = 8.72), and the mean experience in PC was 15.9 years (SD = 12.09) (Table 2).

The codes used in the analysis are illustrated by textual quotations from the participants (verbatims). Participants verbatims were identified by their code, age, sex (M = Men/W = Woman), and whether they had participated in the INCOPAL project (yes/no).

### 3.1. Palliative Needs

**Figure 1:**
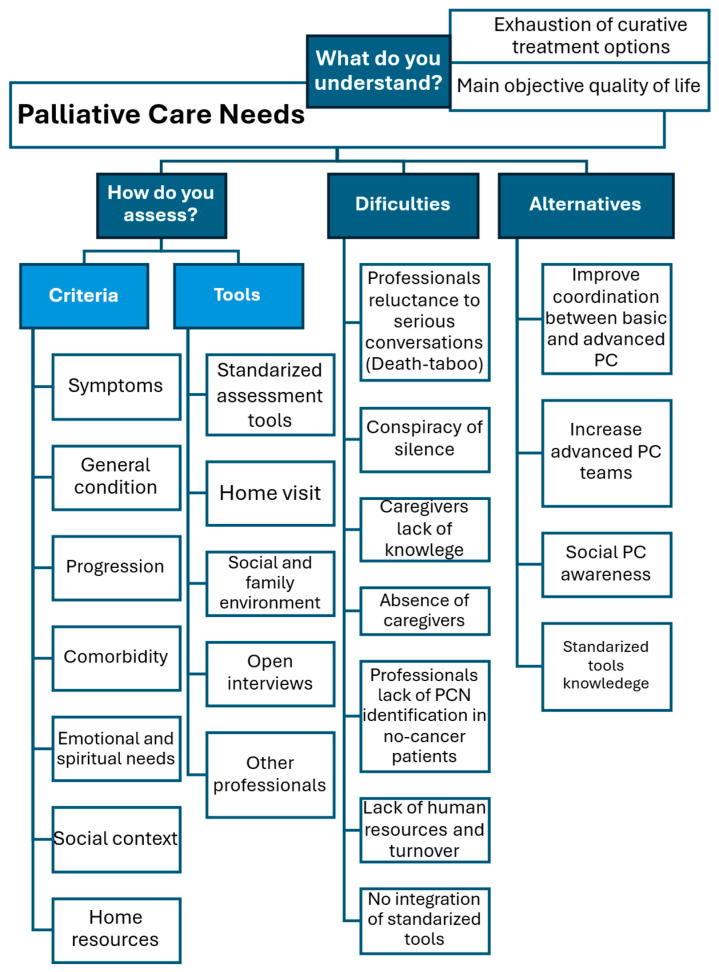
Palliative Care Needs codes, themes and sub-themes.

The codes, themes and sub-themes related to PCNs are shown in Figure 1.

#### 3.1.1. What Do You Understand by PCNs?

Although none of the participants clearly defined what they understood by PCN, most agreed that patients with PCN are those with cancer or non-cancer diseases who have *exhausted all curative treatment options*. At this point, the *main objective becomes the quality of life* of both the patient and their family. One interviewee emphasized that PC should begin as soon as an incurable disease is diagnosed, without waiting for the patient to reach the terminal stage.

“You are going to focus on comfort care, on care that does not go beyond simply making the patient feel as comfortable as possible.” (19, 49 y, W, No INCOPAL)

“Above all, you have to provide care, comfort measures aimed more at their well-being, more at the intention of caring than at curing those diseases.” (4, 48 y, W, No)

“Patients with complex chronic conditions that progress to the end and patients who have cancer, where the progression is much faster and more aggressive, and who may come to me at much more complex stages.” (16, 55 y, W, No)

“Palliative care should be provided from the moment I am diagnosed with an incurable disease.” (2, 30 y, M, No)

#### 3.1.2. How Do You Assess PCNs?

The assessment of PCN involves a comprehensive evaluation of different *criteria*: clinical *symptoms*, the patient’s *general condition*, their *progression*, and the presence of *comorbidities*. In this assessment, the *emotional and spiritual needs* of patients also become relevant.

“Assessing not only clinical and care aspects, but also emotional and spiritual aspects is essential because, at the end of the day, is what moves us, and what perhaps enables us to face these difficult moments.” (10, 57 y, W, yes)

“Above all, addressing those spiritual needs of how they are coping with the process, what strengths they have as internal resources when working through moments of crisis in their daily lives.” (16, 55 y, W, No)

Regarding PCNs assessment tools, some interviewees use *standardized assessment tools* that are integrated into the computer system in their daily practice. The NCMs participating in the INCOPAL project mention that they use different instruments to identify PCN and the IDC-pal to determine the degree of complexity of the patient.

“At the clinical level, I assess palliative needs first according to clinical symptoms, clinical pathology, progression, patient dependence, and the complexity of that care.” (1, 46 y, W, yes)

“Through the IDC-pal and the Barthel, Pfeiffer, social, and Gijón questionnaires. And if I see that the patient is in the final stage, the Karnofsky as well” (17, 50 y, W, No)

Some interviewees highlighted the importance of assessing not only the patient, but also their *social and family environment*, especially caregivers burden and the availability of resources in the home.

“Knowing what the family environment is like, what problems they have, what that patient means to the family, well, that’s very important in order to care both, the patient and the family.” (8, 57 y, W, yes)

“I don’t just focus on the person. I also focus on the caregiver, the family surrounding the palliative patient, and what I do is an assessment of basic needs.” (18, 56 y, W, No)

In this sense, *home visits* are essential for assessing PCN. *Open interviews* with the patient allow them to express their concerns and needs more freely, obtaining information that is not always revealed in a standard consultation with a structured interview.

“I usually try to listen a lot, listen carefully to what they tell me because sometimes it gives me much more information than when you are interrogating them.” (7, 61 y, W, yes)

“I like it that way, directly with the patient and in a natural way, which is good. Which is part of the process, of course.” (2, 30 y, M, No)

On the other hand, some interviewees point out that they do not have the opportunity to directly evaluate PCNs, as these are assessed in advance by *other healthcare professionals* before their intervention.

“They usually come already defined by the internist.” (12, 54 y, W, No)

“I access palliative care patients through hospital NCMs and through my colleagues, so they practically come to me already assessed.” (2, 30 y, M, No)

#### 3.1.3. Difficulties in Assessing PCNs

Among the difficulties in assessing PCN, all participants highlighted the *conspiracy of silence*, which limits open communication about the reality of the disease and hinders informed decision-making. Healthcare professionals themselves contribute to this situation by avoiding difficult conversations about prognosis and limited treatment options.

“I try to make the family understand that I know that they want to protect their relative (…) But I also try to make them see that the patient has rights and we cannot deny them; Patients have the right to know or not to know.” (18, 56 y, W, No)

“Often, this conspiracy of silence comes from professionals who do not dare to communicate the few therapeutic options available and show a difficult reality.” (10, 57 y, M, yes)

Some interviewees acknowledge that *caregivers do not have the adequate knowledge* to take on the task of caregiving. One participant mentions the progressive decline in informal caregivers or *absence of caregivers* due to changes in family structure.

“Sometimes what I find is that you have to inform the family on how to use all the resources they may have.” (12, 54 y, W, No)

“There are not as many caregivers available anymore. Caregivers are older, they are also very advanced in age.” (10, 57 y, M, yes)

Almost all interviewees agree that *death remains a taboo* in society, which makes it difficult to accept PC. There is still *professionals’ reluctance* to address this type of care, perceiving it as synonymous with failure. Family members and patients also share this perception.

“The society we live turns its back on death. It is a society that only seeks to extent life at any price.” (16, 55 y, W, No)

“Culturally, when you reach palliative care, it is a failure. In fact, patients and family members experience it that way at first.” (15, 60 y, W, yes)

On the other hand, the *lack of training in PC* is another significant barrier that hinders the identification and addressing of patients’ needs. This lack of training particularly impacts professionals’ ability to address patients’ emotional and spiritual needs. In addition, there is a notable *lack of training in identifying PCN in non-oncology patients*, which contributes to many patients having delayed access to PC, limiting the interventions and support they can receive.

“There are areas that we tend to neglect, which, in the context of the end of life, need to be worked on, such as the emotional and spiritual spheres, which are difficult to work on with patients and which we find a little difficult to address.” (4, 48 y, M, No)

“We all understand that when we cannot cure a cancer patient, they are palliative, but what about those frail elderly people, those with heart failure, those with COPD...” (6, 34 y, W, No)

“I see that these patients continue to attend consultations, follow-ups, and appointments with specialists, and no one makes the decision to say that at this point the patient is in a palliative situation.” (19, 49 y, W, yes)

Participants also pointed out the *lack of healthcare personnel* and the continuous *turnover* of staff, especially nurses. Short-term contracts create instability in teams, which affects continuity of care and hinders specialization in PC. In addition, they cite a lack of time to perform a comprehensive assessment of patients, which leads them to prioritize the most obvious cases. All of this generates frustration and helplessness among professionals.

“The fundamental problem is the mobility we have had lately with professionals” (19, 49 y, W, yes)

“I don’t know if in primary care, healthcare professionals are able to devote time, and perhaps that is what is failing us in identifying those patients who are not affiliated with palliative care.” (6, 34 y, W, No)

The NCMs participating in the INCOPAL project indicated that essential tools, such as the IDC-Pal or NECPAL, are *not integrated into the computer system*, which makes it difficult to apply and record them in the medical record. Furthermore, the medical record is not always completed properly, and information regarding previous interventions, referrals, and home visits is not clearly reflected.

“The computer tool is used here in Andalusia, but it doesn’t have many tools that facilitate that assessment.” (4, 48 y, M, No)

“It’s true that we have the IDC-Pal scale, but that scale, for example, is not registered in (the computer system). Since we don’t have it registered, it’s more complicated to register it.” (11, 60 y, W, No)

#### 3.1.4. Alternatives to Actual PCNs Assessment

As alternatives for assessing PCN, some interviewees highlighted the need to *improve coordination basic and advanced level of PC*, that might enhance continuity of care. Others, however, propose *increasing the number of specialized PC nurses and doctors*. One participant suggests implementing a “red button” in the computer system that would allow for early identification of patients with PCN, even before they are referred to specific resources.

“We should all work together so that the patient really benefits from my timely intervention, but also from the continuity of care provided by the palliative care unit and primary care.” (6, 34 y, W, No)

“Provide palliative care with more staff so that they can care for patients entering the palliative process, regardless of the level of complexity.” (17, 50 y, W, No)

“That you can really identify it in (computer system), medicine, and nursing, even if you can’t refer it to the teams.” (8, 57 y, W, yes)

Several interviewees highlighted the need to *raise awareness* among both professionals and society about the importance of PC. This involves ensuring access to PC training for all professionals. Some suggested that training should be mandatory during working hours. Interviewees who did not participate in the INCOPAL project emphasized the *need to know scales and instruments for assessing PCNs*, while those who collaborated in the project highlighted that it has allowed them to learn about and become familiar with many of these tools.

“End of life is part of the life cycle and that professionals have to be prepared to respond to that situation and also adapt and establish strategies for action.” (6, 34 y, W, No)

“Perhaps offering some kind of more advanced course that people could take at a more affordable price and that would also be easier to attend in terms of work.” (7, 61 y, W, yes)

### 3.2. Referral to PC

**Figure 2:**
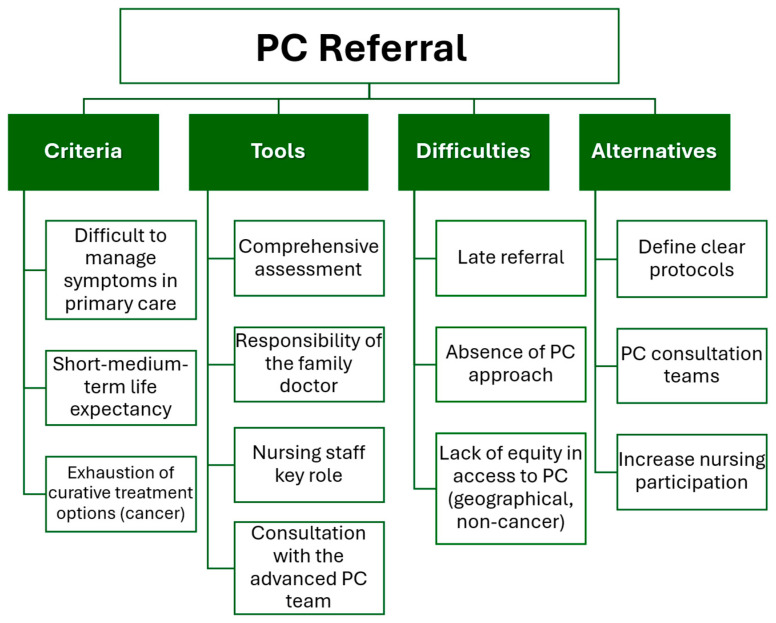
Referral to advanced PC resources codes, themes and sub-themes.

The themes and sub-themes related to referral to advanced PC resources are shown in Figure 2.

#### 3.2.1. Criteria for Specialized PC Referral

One of the criteria for referral to PC most frequently mentioned by participants is the presence of *symptoms that are difficult to manage in primary care setting*. Participants emphasized that these patients require specialized management due to the complexity of their needs.

“When I make that assessment and find that the patient is highly complex and beyond the response we can give in primary care, that’s when I refer them.” (18, 56 y, W, No)

“Those complex chronic patients who are basically immobilized with almost 24-h home care. That’s when I understand that it’s time to move the person into the palliative care process.” (10, 57 y, M, yes)

Other interviewees point out that referral to PC is considered when the patient is estimated to have a *short- or medium-term life expectancy*. Likewise, other interviewees state that, in their experience, referral to PC is usually made for cancer patients who cannot tolerate treatment and have *exhausted all therapeutic options*.

“If the person is in a palliative situation, undergoing palliative treatment, with no cure, and it is a matter of time and a short- to medium-term prognosis, then palliative care should be initiated.” (9, 57 y, W, No)

“When I see that the situation is more complex and when the patient requires, let’s say, a short-term prognosis.” (11, 60 y, W, No)

“When the signs and symptoms treated by their family doctor do not resolve the situation or improve the patient’s condition, we have exhausted all resources in terms of treatment, tests, and so on. Then we need more advanced resources.” (13, 61 y, W, yes)

#### 3.2.2. Tools for Specialized PC Referral

In relation to the referral process, the first step is to perform a *comprehensive assessment*. Half of the participants agreed that this assessment is the *responsibility of the family doctor*, although *nursing staff play a key role*. Several interviewees emphasized that this process requires close coordination with the multidisciplinary team.

“ The doctor performs a physical examination and so on. While we make our assessment based on needs and also perform a physical examination.” (18, 56 y, W, No)

“Talking to the care team: family doctor, family nurses, social worker at the healthcare centre, in order to communicate the situation of reversibility, worsening, poor prognosis, increased intensity of interventions and visits...” (10, 57 y, M, yes)

Many of the participants highlighted the *consultation with the specialized PC team*, emphasizing the need for fluid communication. Other NCMs stated that, in some cases, it is the family who requests PC when they perceive a deterioration in the patient’s condition.

“If a patient is referred who is in a much more advanced stage and I want them to be seen soon, I speak to them (specialized PC team) directly and maybe they will see them the next day.” (14, 60 y, W, yes)

“Often it is the family who contacts you, because sometimes you don’t get to see all the patients, they are being seen by specialists.” (20, 56 y, W, yes)

#### 3.2.3. Difficulties for Specialized PC Referral

Among the difficulties in referral to PC, some interviewees pointed out that referral often occurs *too late*, when the patient is already in the last days of life. They mentioned that, in practice, it is difficult to refer patients in the early or intermediate stages of the process, due to the *reluctance of certain health professionals to adopt a palliative care approach*.

“They act when the patient is already in a very advanced or final stage, when it is time to sedate, while all the literature tells you is that these teams have to work intensively at the beginning of the process.” (8, 57 y, W, yes)

“They want to be too clear when sending patients to palliative care. It has to be too obvious from the beginning to send them.” (15, 60 y, W, yes)

On the other hand, participants highlighted the *lack of equity in access to PC*, depending on *geographical factors*. Some patients from rural areas die without receiving adequate PC, far from their families or without the possibility of staying at home, as they would wish. Several interviewees emphasized that *non-cancer patients* have more difficult access to PC.

“It is very sad that, depending on where you live, you may die with more or less dignity because if you want to die at home, if you don’t have a home support and palliative care team, you may not be able to consider it.” (14, 60 y, W, yes)

“I mean that it (PC) depends on the professional you get, it depends on which hospital you are in, it depends on which service, it depends on which health centre your home is in, it depends on whether you get the morning shift, the afternoon shift, the night shift...” (18, 56 y, W, No)

Some participants suggested the need to *define clear protocols* for referring patients to the hospital, avoiding unnecessary steps, and optimizing coordination between PC and hospital services. Other NCM suggested the creation of *PC consultation teams* within districts or hospital centres, to offer advice and continuous updating on the management of PC patients. Several interviewees recommended that referral to PC should not depend exclusively on the physician, but that the *nursing team should actively participate* in the referral and decision-making process. One interviewee proposed the creation of a specialized team for non-oncological diseases, and another the creation of a specific form in the computer system.

“Establishing this formal, well-known, and clear circuit, especially in the event that a patient needs to be referred to a hospital. Above all to avoid that initial hassle at the emergency room door.” (4, 48 y, M, No)

“It would be great to have a unit or a team of highly trained people in the district or hospital or somewhere where we can ask for information or where we can always be up to date.” (2, 30 y, M, No)

“There should also be an advanced support team to help us primary care professionals carry out this follow-up and approach work for non-cancer patients.” (10, 57 y, M, yes)

“Nurses are the ones who are actually doing the work at home, and follow-up care for patients at home is mainly carried out by nurses, which means that nurses are the first to realize when a patient needs palliative care.” (16, 55 y, W, No)

“If there was a questionnaire or form in the computer program that could be filled out and sent to palliative care at to be viewed, it would be much faster” (17, 50 y, W, No)

### 3.3. Evaluation of the INCOPAL Project

#### 3.3.1. Changes in Participants’ Understanding of PC

The INCOPAL project has generated changes in the perception and approach to PC by the participating NCMs. It has allowed them to *understand that the approach to PC* transcends physical symptoms, and they have adopted a *more holistic view* in their professional practice. One of the participants stated that his perception of which patients require PC has changed, now *including non-oncology patients*. Another interviewee mentioned that the project has helped to highlight the *importance of early detection of PCN*, allowing for better care planning.

“What really needs to be identified is not just the symptoms, but much more than that, namely the families, the team, how to approach that patient with the entire team.” (1, 46 y, W, yes)

“We always have the concept of palliative care for cancer patients, and really, with this participation, I have seen that this is not the case. Palliative patients involve many more pathologies than just cancer.” (1, 46 y, W, yes)

“The project helps you to appreciate more or make more visible the palliative care needs at an earlier stage.” (15, 60 y, W, yes)

Some NCM stated that the project has made them *more aware of the difficulties in PC*, such as the lack of resources or the difficulty of accessing the services. Others highlighted the *importance of scales and questionnaires* to assess patients in a more accurate and structured way.

#### 3.3.2. Criticism of INCOPAL Project

In this regard, some of the participants recommended *reducing the number of questionnaires*, eliminating those that are redundant or irrelevant, or combining the questionnaires into one. On the other hand, one participant pointed out that he has missed *specific instruments to assess the situation of the primary caregiver*.

“It really reflects the gap between palliative needs and the response we provide.” (8, 57 y, W, yes)

“Knowing some questionnaires and assessment scales that we could incorporate into that assessment to make it more complete.” (13, 61 y, W, yes)

“There are questionnaires that I would remove because an assessment, an evaluation of the patient with all these questionnaires is practically impossible, so I would reduce them.” (1, 46 y, W, yes)

“There are no scales aimed at assessing the caregiver’s situation, the caregiver’s emotions, the difficulty the caregiver has had or is having in caring for that complex chronic person in palliative care. I have missed them.” (10, 57 y, M, yes)

## 4. Discussion

This is one of the first studies to describe the experience of NCM in palliative care needs identification and CCP referral to advanced PC resources.

The NCMs interviewed conduct a comprehensive assessment of PCN using standardized scales and open interviews as assessment tools. Among the criteria for referral to advanced PC resources, are difficult-management symptoms and limited prognosis. Barriers such as the conspiracy of silence, lack of training in specialist care, and scarcity of resources persist, leading to late referrals and inequality in access to specialist care.

Late identification of PCN is one of the main difficulties in providing PC. PCN are often recognized too late, limiting the possibility of early intervention in the course of the disease. In this regard, the participants in our study indicated that referral to PC takes place when the available therapeutic options have been exhausted. Only one of them pointed out that PC should begin at the onset of the life-threatening disease.

According to Van der Stap et al. [14], curative and disease-focused treatments are often prolonged excessively, delaying the initiation of PC and negatively impacting the quality of care. Conversely, the implementation of early palliative care improves the patient’s understanding of their disease process and the quality of end-of-life care and reduces the use of excessive therapeutic measures during the last month of life [15]. The regular use of standardized tools such as NECPAL [5] in CCP can improve access to PC for these patients.

One of the problems pointed out by participants is the lack of attention to non-cancer patients. PC was originally designed for terminally ill cancer patients, so there is limited knowledge about when and how to transition to PC in non-cancer patients [16]. Bonares et al. [17] demonstrated, in a comparison between cardiologists, pulmonologists, and oncologists in Canada, that non-cancer patients are less likely to receive specialized palliative care and are referred later. In this regard, Jang et al. [18] show that non-cancer patients may experience levels of suffering equal to or even greater than cancer patients, due, among other factors, to the lack of identification of PCN in these patients.

The lack of human and material resources to perform a comprehensive assessment of the patient in PC negatively impacts the assessment of PCN. Pitzer et al. [19] identified in a mixed methods systematic review that lack of human resources is a main barrier in providing PC. These difficulties were supported by Jones et al. [20], who highlighted the shortage of nurses in PC, as well as geographical inequality in access to specialized PC services. Future studies should consider staffing levels and the geographical dispersion of the population as key factors of inequality in access to PC in cancer and non-cancer patients.

The healthcare professionals interviewed reported lack of training in PC, especially in addressing patients emotional and spiritual needs. A recent review [21] highlighted that PC professionals face a lack of training and insufficient preparation to deliver spiritual care. Pavlic et al. [22] supported this observation by pointing out that, while pain management is usually include in healthcare training, other fundamental aspects, such as non-physical symptoms and the psychosocial and spiritual dimensions of care, are often insufficiently addressed, especially in non-cancer patients.

This research shows that some professionals continue to perceive PC as synonymous with therapeutic failure. The focus on cure, the nonacceptance of terminal prognosis and the negative perceptions of palliative care was highlighted as a barrier in the provision of PC in Pitzer et al. review [19]. Shen and Wellman [23] pointed to the existence of a social stigma associated with PC, linking them to surrender, weakness, or the loss of hope, which influences people’s willingness to accept them for themselves or their loved ones.

### 4.1. Strengths and Limitations

This study is one of the first studies conducted to explore the experience of NCMs in PCN identification and CCP referral to advanced PC resources. The inclusion of professionals from different parts of Andalusia is a strength, as it provides a diversity of perspectives; however, the uneven distribution of the sample across provinces may have influenced the results, given that working conditions and the organization of health services vary considerably across the region. The majority participation of female NCMs accurately reflects the feminization of the nursing profession, providing a realistic picture of the professional profile in PC. However, this overrepresentation may have partially conditioned the interpretation of the results, limiting the male perspective. Finally, although the cross-sectional design captures the perceptions of NCMs at a specific point in time, it offers an up-to-date snapshot of the impact of the INCOPAL project on clinical practice. As a qualitative study, the results should be considered carefully to extrapolate conclusions to other similar contexts. Nevertheless, qualitative research methodology does not aim for representativeness of the results, but rather for understanding the phenomena.

### 4.2. Clinical, Policies and Research Implications

From a clinical perspective, the systematic use of validated tools such as NECPAL [5] should be promoted to enhance the early detection of PCN and facilitate proactive care planning in CCP. Policymakers should prioritise the incorporation of standardized instruments into digital systems and ensure time for comprehensive assessment.

Furthermore, strengthening NCM competencies through structured PC education programs is crucial, particularly in areas such as psychosocial and spiritual care.

Clinical practice should also prioritize the inclusion of non-cancer patients, who continue to experience inequitable access to PC. Policies should promote disease-specific referral criteria and interprofessional protocols, particularly those that recognise nurses’ competencies in initiating referrals, could mitigate this imbalance and ensure equitable PC delivery across diagnostic groups.

Research should explore interventions aimed at reducing professional barriers to PC implementation and examine their impact on clinical decision-making and patient satisfaction.

Finally, policymakers and managers should address structural determinants of inequality in PC, including workforce shortages, regional disparities, and limited service availability. Addressing these gaps will be essential to advancing equitable, holistic, and patient-centred PC for all individuals with life-limiting illnesses.

## 5. Conclusions

For NCMs, early comprehensive assessment of PCN in oncological and non-oncological patients and their families is a key element of PC in primary care. NCMs use standardized scales, home visits, and open interviews, and they face barriers such as the conspiracy of silence, death taboo, lack of training and human resources. Difficult-to-manage symptoms and limited prognosis are prominent factors in the referral of CCPs to PC. Late referral and inequity in access to advanced PC resources continue to occur due to geographical dispersion and limited visibility of the needs of non-oncological patients. It is necessary to strengthen coordination between levels of care, establish clear protocols, and enable digital tools that facilitate a more agile and equitable response to PC.

## Figures and Tables

**Table 1:** Semi-structured interview questions.

	Questions
Palliative Care Needs	What do you understand by palliative needs? How do you assess palliative needs in your daily practice? What difficulties do you face in assessing palliative needs? What would you change? What alternatives would you have?
PC Referral	When do you refer a case to palliative care? What is that process like? What difficulties do you face in referring to palliative care? What would you change? What alternatives would you have?
INCOPAL Evaluation	How has your view of palliative care needs assessment and referral to palliative care changed because of participating in the project?

**Table 2:** Participants characteristics.

	INCOPAL	Age (Years)	Gender	Experience in Primary Care (Years)
1	Yes	46	Female	10
2	No	30	Male	7
3	Yes	61	Male	39
4	No	48	Male	9
5	No	46	Female	3
6	No	34	Female	4
7	Yes	61	Female	35
8	Yes	57	Female	18
9	No	57	Female	21
10	Yes	57	Male	35
11	No	60	Female	20
12	No	54	Female	3
13	Yes	61	Female	5
14	Yes	60	Female	22
15	Yes	60	Female	7
16	No	55	Female	3
17	No	50	Female	20
18	No	56	Female	34
19	No	49	Female	17
20	Yes	56	Female	6

## Data Availability

The database used and analyzed in this study is available from the corresponding author upon reasonable request. Access is restricted due to confidentiality, privacy, and ethical considerations, as the data contain sensitive personal information that could potentially identify study participants.

## References

[1] Palliative Care-World Health Organization. https://www.who.int/es/news-room/fact-sheets/detail/palliative-care.

[2] Blay C., Limón E. (2017). Bases Para un Modelo Catalán de Atención a las Personas con Necesidades Complejas: Conceptualización e Introducción a los Elementos Operativos. Programa de Prevenció i Atenció a la Cronicitat.

[3] Ministerio de Sanidad, Servicios Sociales e Igualdad (2012). Estrategia Para el Abordaje de la Cronicidad en el Sistema Nacional de Salud.

[4] Amblàs-Novellas J., Murray S.A., Oller R., Torné A., Martori J.C., Moine S., Latorre-Vallbona N., Espaulella J., Santaeugènia S.J., Gómez-Batiste X. (2021). Frailty degree and illness trajectories in older people towards the end-of-life: A prospective observational study. BMJ Open.

[5] Gómez-Batiste X., Martínez-Muñoz M., Blay C., Amblàs J., Vila L., Costa X., Espaulella J., Villanueva A., Oller R., Martori J.C. (2017). Utility of the NECPAL CCOMS-ICO© tool and the Surprise Question as screening tools for early palliative care and to predict mortality in patients with advanced chronic conditions: A cohort study. Palliat. Med..

[6] Calsina-Berna A., AmblàsNovellas J., González-Barboteo J., Bardés Robles I., Beas Alba E., Martínez-Muñoz M., Madariaga Sánchez R., Gómez Batiste Alentorn X. (2022). Prevalence and clinical characteristics of patients with Advanced Chronic Illness and Palliative Care needs, identified with the NECPAL CCOMS-ICO© Tool at a Tertiary Care Hospital. BMC Palliat. Care.

[7] World Health Organization (2018). Integrating Palliative Care and Symptom Relief into Primary Health Care: A WHO Guide for Planners, Implementers and Managers.

[8] Radbruch L., Payne S. (2009). White paper on standards and norms for hospice and palliative care in Europe: Part 1. Eur. J. Palliat. Care.

[9] Martín-Roselló M.L., Fernández-López A., Sanz-Amores R., Gómez-García R., Vidal-España F., Cía-Ramos R. (2014). IDC-Pal: Instrumento Diagnóstico de la Complejidad en Cuidados Paliativos. Documento de Apoyo al PAI Cuidados Paliativos.

[10] Carrasco-Zafra M.I., Gómez-García R., Ocaña-Riola R., Martín-Roselló M.L., Blanco-Reina E. (2020). Level of Palliative Care Complexity in Advanced Cancer Patients: A Multinomial Logistic Analysis. J. Clin. Med..

[11] Junta de Andalucía, Consejería de Salud y Consumo (2019). Proceso Asistencial Integrado: Cuidados Paliativos.

[12] Husserl E. (1998). Invitación a la Fenomenología.

[13] Sandelowski M. (2010). What’s in a name? Qualitative description revisited. Res. Nurs. Health.

[14] Van der Stap L., de Nijs E.J.M., Oomes M., Juffermans C.C.M., Ravensbergen W.M., Luelmo S.A.C., Horeweg N., van der Linden Y.M. (2021). The self-perceived palliative care barriers and educational needs of clinicians working in hospital primary care teams and referral patterns: Lessons learned from a single-center survey and cohort study. Ann. Palliat. Med..

[15] Hui D., Heung Y., Bruera E. (2022). Timely Palliative Care: Personalizing the Process of Referral. Cancers.

[16] Kim S., Lee K., Kim S. (2020). Knowledge, attitude, confidence, and educational needs of palliative care in nurses caring for non-cancer patients: A cross-sectional, descriptive study. BMC Palliat. Care.

[17] Bonares M., Le L.W., Zimmermann C., Wentlandt K. (2023). Specialist Palliative Care Referral Practices Among Oncologists, Cardiologists, Respirologists: A Comparison of National Survey Studies. J. Pain Symptom Manag..

[18] Jang H., Lee K., Kim S., Kim S. (2022). Unmet needs in palliative care for patients with common non-cancer diseases: A cross-sectional study. BMC Palliat. Care.

[19] Pitzer S., Kutschar P., Paal P., Mülleder P., Lorenzl S., Wosko P., Osterbrink J., Bükki J. (2024). Barriers for Adult Patients to Access Palliative Care in Hospitals: A Mixed Methods Systematic Review. J. Pain Symptom Manag..

[20] Jones R., Dale J., MacArtney J. (2023). Challenges experienced by NCMs when providing palliative care in the UK: A systematic qualitative literature review. BJNCM Open.

[21] Costeira C., Querido A., Ventura F., Loureiro H., Coelho J., Benito E., Nabal M., Dones M., Specos M., Laranjeira C. (2024). Spiritual Care [Givers] Competence in Palliative Care: A Scoping Review. Healthcare.

[22] Rotar Pavlič D., Aarendonk D., Wens J., Rodrigues Simões J.A., Lynch M., Murray S. (2019). Palliative care in primary care: European Forum for Primary Care position paper. Prim. Health Care Res. Dev..

[23] Shen M.J., Wellman J.D. (2019). Evidence of palliative care stigma: The role of negative stereotypes in preventing willingness to use palliative care. Palliat. Support. Care.

